# Less Experienced Telestroke Consultants Are More Likely to Go On-Camera, but Less Likely to Give tPA

**DOI:** 10.1155/2019/1059369

**Published:** 2019-11-13

**Authors:** Adam de Havenon, Lee S. Chung, Jaleen Smith, Kirby Taylor, Jennifer J. Majersik, Nabeel Chauhan

**Affiliations:** Department of Neurology, University of Utah, 175 N Medical Dr E, Salt Lake City, UT 84132, USA

## Abstract

**Background:**

Stroke telemedicine (telestroke) increases tPA availability and administration. However, the effective use of telestroke requires training, which is not a standard component of vascular neurology training. As a result, many providers learn telestroke skills “on the job” after finishing their training.

**Aims:**

We sought to explore if providers with more telestroke experience would be more efficient in the utilization of telemedicine, compared to providers with less experience.

**Methods:**

We prospectively collected data on telestrokes between July 2014 and July 2017 at a Comprehensive Stroke Center. Telestrokes are initiated on the telephone and typically, but not always, followed by an on-camera consult. Decision to do a phone-only versus on-camera consult is at the provider's discretion.

**Results:**

There were 1,029 telestrokes, of which 807 were on-camera (74%). Of the 8 telestroke providers, 4 had less experience, having just finished stroke fellowship, and 4 had more experience (mean = 7.8 years of telestroke experience at the beginning of the study). Providers with less experience were more likely to go on camera than providers with more experience (79% vs. 67% of consults, *p* = 0.021), but were less likely to give tPA when on-camera (25% vs. 33%, *p* = 0.023). The absolute rate of tPA administration, combining phone and camera administration, or the frequency of technical difficulties were not different.

**Conclusions:**

Telestroke consultants with less experience do not triage as many cases by phone and are less likely to administer tPA on-camera, suggesting their use of telemedicine is not optimized. This supports the introduction of telestroke didactics during vascular neurology training.

## 1. Introduction

In 1999 a stroke telemedicine (telestroke) system was first described, which noted that telestroke technology could be a key tool to increase geographical access to tissue plasminogen activator (tPA) [1]. In subsequent prospective studies, telestroke was found to increase tPA administration while also decreasing unnecessary hospital transfers [2, 3]. In 2009, the American Heart Association/American Stroke Association (AHA/ASA) recommended that telestroke be implemented within all stroke systems of care that had gaps in access [4]. Despite significant progress in their availability, telestroke services will need to further expand to address the geographical clustering of neurologists and increasingly complex and resource-intensive stroke care. However, to effectively use telemedicine for stroke requires training, which is not a required component of ACGME-approved vascular neurology fellowships in the United States. As a result, most providers learn to do telestroke after they have graduated from residency or fellowship [5]. We hypothesize that “on the job” telestroke training results in inefficient telestroke utilization during the beginning of vascular neurologists' careers.

## 2. Methods

We prospectively collected data on all telestroke consults performed between July 2014 and July 2017 at a Comprehensive Stroke Center in the United States with a traditional hub and spoke telestroke model. The number of spokes grew during this time period from 16 to 26 hospitals. Telestroke providers were board certified in vascular neurology (*n* = 7), except for one provider that is an experienced neurohospitalist who has been taking telestroke call since the program's inception 15 years prior. All consults are initiated on the telephone by the spoke hospital contacting our transfer center. The telestroke provider is then connected to the spoke hospital physician on the telephone. They discuss the relevant details of the case, such as patient presentation, NIH Stroke Scale, and clinical variables. Typically, but not always, this phone conversation is followed by an on-camera consult. The protocol for telestroke providers indicates that if tPA or another acute intervention such as endovascular thrombectomy is being considered, then they will provide an on-camera consult. Otherwise, the decision to do a phone-only versus on-camera consult is entirely at the discretion of the telestroke provider. With an IRB waiver, we prospectively collected data on all telestroke encounters, including phone-only and on-camera consults. We also collected data on the frequency of technical failures involving telestroke connections and the percentage of cases in which the failure could not be resolved. To address our hypothesis, we dichotomized the consultants based upon level of experience as a telemedicine physician (less versus more experienced providers). Intergroup comparisons were performed in Stata 14.1 using Student's *t*-test, which is robust to small and equal sample sizes.

## 3. Results

**Figure 1:**
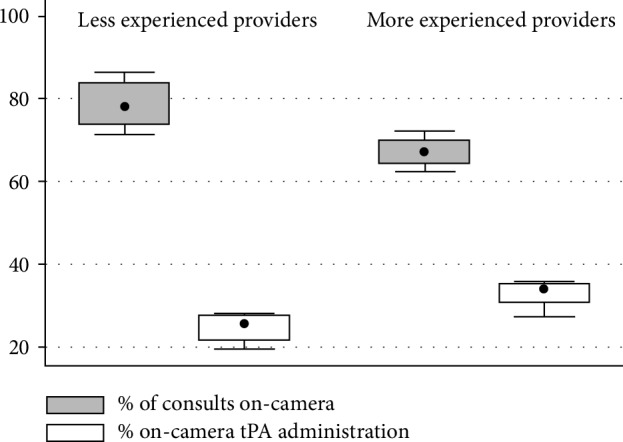
Box-and-whiskers plot showing telestroke consults on-camera and tPA provided on-camera between providers with less and more experience, with box representing interquartile range, dot the median, and whiskers the range.

The telestroke providers performed 1,092 telestroke consults during the specified time period, of which 807/1,092 (73.9%) were on-camera. There were 4 telestroke providers with less experience (all having just finished vascular neurology fellowship at the beginning of the study) and 4 providers with more experience (mean ± SD years = 7.8 ± 3.1) (*p* = 0.002 for difference). Providers with less experience were more likely to go on camera than providers with more experience (79% vs. 67% of consults, *p* = 0.021), but were less likely to give tPA during the on-camera consults (25% vs. 33% of consults, *p* = 0.023) (Figure 1). When considering the absolute number of times tPA was given, which combines both on-camera and phone-only consults, there was no difference in the rate of tPA administration (20% vs. 25%, *p* = 0.128). There was no significant difference in the frequency of technical difficulties, including the percentage of on-camera consults with any technical connection issue or percentage of technical issues that resulted in failure of on-camera consult (Table 1).

## 4. Discussion

In our study of 1,029 telestroke consults at a Comprehensive Stroke Center, providers who recently finished vascular neurology fellowship were more likely to go on-camera than more experienced providers, but were less likely to give tPA on-camera. This suggests that telestroke providers with less experience are spending more time on-camera in an inefficient manner. There are several possible explanations. Less experienced providers may not be comfortable with their phone triage abilities and be wary of missing potential tPA cases. They may be less trusting of outside ED physicians or may be more conscious of spoke site satisfaction. Although we hypothesized that telestroke providers with less experience would experience more technical difficulties and problems resolving connection issues, we did not find a significant difference in these outcomes based on provider experience.

Since the early 2000s the rate of administration of tPA for acute ischemic stroke has nearly doubled [6]. Telestroke has contributed to this rise by increasing geographical access to vascular neurology consultation [7]. The functional neurologic outcome and mortality rate between telestroke-guided tPA administrations at spoke hospitals versus tPA administrations at stroke hub centers are comparable [8]. However, stroke patients who present to rural hospitals can be up to ten times less likely to receive tPA than those at urban hospitals [9]. The AHA/ASA formally recommends telestroke consultation be available and guidelines from the American Telemedicine Association suggest the incorporation of neurologists in training [10, 11]. Despite these recommendations and the proven effectiveness of telestroke consultation, telestroke training is not a standard component of vascular neurology fellowships and there is no formal curriculum or milestones to guide the training for acute stroke consultations via telemedicine. Likewise, there is no curriculum addressing the complexity of the hub and spoke model and associated administrative and technological infrastructure. As a result, many providers learn telestroke skills after finishing their training. A previously reported retrospective study found that the time from stroke onset to tPA bolus was longer for vascular neurology fellows than vascular neurology attendings, but the time difference did not result in worse outcomes [12].

Our study has the strength of a large number of telestrokes, prospective data collection, and two groups of telestroke providers with distinct levels of experience. There are also several limitations. The first is that we are unable to explore the reasons for phone-only consults, as they are not documented in our medical record. These data would be important to better understand why more experienced telestroke providers decided to forgo on-camera consults. We do not have a rigid protocol for the initiation of telestroke, which is begun by the outside ED physician. As such, we do not track deviations from the protocol or reasons why telestroke providers did not go on-camera, which are numerous, ranging from patients being outside the tPA window to having nonstroke pathology such as seizure. Without these data, we cannot exclude an underlying imbalance in the pathology or time from last known normal. We also do not know if some of the phone-only consults received tPA. However, given our policy of going on-camera for all treatment decisions like tPA, this should be a small number of cases. We also did not record time intervals from telestroke page or on-camera time to tPA administration, which would have been an interesting data point to compare based on provider experience.

We present novel findings that are relevant to the incorporation of telestroke training into vascular neurology fellowships. We found that telestroke consultants with less experience do not triage as many cases by phone and are less likely to administer tPA on camera, suggesting their use of telemedicine is not optimized. This supports the concept of formalized didactic and experiential telestroke training during vascular neurology fellowship.

## Figures and Tables

**Table 1:** Telestroke characteristics between providers with less (all new to telestroke) and more experience (mean = 7.8 years).

Variable (mean ± SD)	Providers with less experience	Providers with more experience	*p*-value for difference of means
Total telestroke consults (phone-only and on-camera) (*n*)	163 ± 22	110 ± 20	0.013
Total consults completed on-camera (%)	78.8 ± 6.4	67.1 ± 4.0	0.021
On-camera consults with any technical connection issue (%)	15.6 ± 3.3	19.4 ± 1.3	0.601
Technical issues that resulted in failure of on-camera consult (%)	2.7 ± 0.8	3.8 ± 2.4	0.407
On-camera consults that resulted in tPA administration (%)	24.7 ± 3.9	33.0 ± 3.8	0.023
Total consults that resulted in tPA administration (%)	19.3 ± 2.5	22.2 ± 3.3	0.217

## Data Availability

Upon reasonable request, the data will be made available by contacting the University of Utah Stroke Center.
